# Comparison of Burrows-Wheeler Transform-Based Mapping Algorithms Used in High-Throughput Whole-Genome Sequencing: Application to Illumina Data for Livestock Genomes^1^

**DOI:** 10.3389/fgene.2018.00035

**Published:** 2018-02-26

**Authors:** Brittney N. Keel, Warren M. Snelling

**Affiliations:** USDA, Agricultural Research Service, U.S. Meat Animal Research Center, Clay Center, NE, United States

**Keywords:** whole-genome sequencing, mapping algorithm, mapper comparison, genomic coverage, livestock

## Abstract

Ongoing developments and cost decreases in next-generation sequencing (NGS) technologies have led to an increase in their application, which has greatly enhanced the fields of genetics and genomics. Mapping sequence reads onto a reference genome is a fundamental step in the analysis of NGS data. Efficient alignment of the reads onto the reference genome with high accuracy is very important because it determines the global quality of downstream analyses. In this study, we evaluate the performance of three Burrows-Wheeler transform-based mappers, BWA, Bowtie2, and HISAT2, in the context of paired-end Illumina whole-genome sequencing of livestock, using simulated sequence data sets with varying sequence read lengths, insert sizes, and levels of genomic coverage, as well as five real data sets. The mappers were evaluated based on two criteria, computational resource/time requirements and robustness of mapping. Our results show that BWA and Bowtie2 tend to be more robust than HISAT2, while HISAT2 was significantly faster and used less memory than both BWA and Bowtie2. We conclude that there is not a single mapper that is ideal in all scenarios but rather the choice of alignment tool should be driven by the application and sequencing technology.

## Introduction

Ongoing developments and cost decreases in next-generation sequencing (NGS) technologies have led to an increase in their application, which has greatly enhanced the fields of genetics and genomics. The evolution of NGS has been paralleled by the development of algorithms to analyze the increasing quantity of data being produced. A fundamental step in the analysis of NGS data is the mapping of the sequence reads onto a reference genome. Efficient alignment of reads onto the reference genome with high accuracy is very important because it determines the global quality of downstream analyses.

Currently, more than 60 different algorithms exist for mapping sequence reads to a reference genome (Fonseca et al., 2012). Most alignment algorithms rely on the construction of auxiliary data structures, called indices, which are made for the sequence reads, the reference genome sequence, or both. Mapping algorithms can largely be grouped into two categories based on properties of their indices: algorithms based on hash tables, and algorithms based on the Burrows-Wheeler transform (BWT; Li and Homer, 2010). Due to their computational efficiency, BWT-based algorithms have become increasingly popular (Zhang et al., 2013). Algorithms based on BWT align entire reads against substrings sampled from the reference genome. Rapid read searching is enabled by storing all of the suffixes of the reference genome sequence using a representation of a data structure called a suffix/prefix trie. BWT (Burrows and Wheeler, 1994), a reversible data compression algorithm, is utilized in conjunction with the Ferragina-Manzini index (FM index; Ferragina and Manzini, 2000) to reduce the memory occupied by the prefix/suffix trie.

Selection of an appropriate alignment tool for NGS data can be a difficult task due to the wide range of available algorithms. There are several factors that influence the performance of aligners, including, but not limited to sequence read length, sequence quality, and sequencing error rate. Additionally, depth of sequencing directly influences the computational efficiency, i.e., execution time and memory consumption. In this study, we focus on the comparison of BWT-based mappers in the context of paired-end Illumina whole-genome sequencing of livestock genomes. The Burrows-Wheeler Aligner (BWA; Li and Durbin, 2009) and Bowtie2 (Langmead and Salzberg, 2012) have been utilized in a large number of livestock studies. We tested these two mappers and HISAT2 (Kim et al., 2015), a newly released software, using simulated sequence data sets with varying sequence read lengths, insert sizes, and levels of genomic coverage. The mappers were evaluated based on two criteria: (1) computational resource and time requirements, and (2) robustness of mapping through the evaluation of precision, recall, and the area under the precision-recall curve. Additionally, computational resource and time requirements were evaluated on several real sequenced genomes with varying sequencing parameters. To our knowledge, this is the first evaluation of HISAT2 applied to whole-genome sequence data.

## Materials and methods

### Data sets

Real and simulated data sets were used in this study. Simulated data were generated from chr1 of the Sscrofa 11.1 genome build [GenBank: NC_010443.5] using DWGSIM (Version 0.1.11; http://sourceforge.net/projects/dnaa/). Twelve paired-end sequence data sets were generated using read lengths and insert sizes commonly used with the Illumina technology (Table 1): read lengths 100 and 150 and insert sizes 350 and 550 bp (with a standard deviation of 10% of the insert size for each data set). The default per base sequencing error rate of 0.02 was used in all simulations. For each read length/insert size pairing three data sets, each comprised of 50 samples (Table S1), were generated based on sequencing depth: high (10x−25x coverage), medium (5x−10x coverage), and low (1x−5x coverage). For each of the data sets, the sequencing depth of each sample was chosen uniformly at random within the coverage bounds. For each sample, DWGSIM generates reads that should map to the reference genome, as well as random reads that should not be mapped. Reads from all simulated data sets were mapped to chr1 of the of the Sscrofa 11.1 genome assembly.

**Table 1:** Parameters for simulated data sets used in this study.

**Data Set**	**Insert size (bp)**	**Read length (bp)**	**Genomic coverage**
H350_100	350	100	High (10x−25x)
H350_150	350	150	High (10x−25x)
H550_100	550	100	High (10x−25x)
H550_150	550	150	High (10x−25x)
M350_100	350	100	Medium (5x−10x)
M350_150	350	150	Medium (5x−10x)
M550_100	550	100	Medium (5x−10x)
M550_150	550	150	Medium (5x−10x)
L350_100	350	100	Low (1x−5x)
L350_150	350	150	Low (1x−5x)
L550_100	550	100	Low (1x−5x)
L550_150	550	150	Low (1x−5x)

In addition to the simulated data sets, we incorporated five real data sets (R1–R5), each comprised of five animals; two sets from cattle and three from swine (Table 2). Data from sets R1 and R4 have been previously described in Keel et al. (2017) and Snelling et al. (2015), respectively. Genomic DNA was extracted from semen or blood using one of the following standard DNA extraction protocols: phenol-chloroform extraction, salt extraction, a QIAamp DNA Mini kit (Qiagen, Germantown, MD) or a Wizard SV96 Genomic Purification kit (Promega Corp., Madison, WI, USA). Genomic DNA was sheared to 350–550 bp, and libraries prepared using either the Agilent SureSelect Target Enrichment System Kit I or Kit II (Agilent Technologies Inc., Santa Clara, CA), the TruSeq DNA sample prep kit, version 2 (Illumina, San Diego, CA), or the TruSeq DNA PCR-Free sample prep kit (Illumina, San Diego, CA). Libraries were paired-end sequenced using either an Illumina HiSeq2500 or NextSeq500 instrument. Reads from data sets R1, R2, and R3 were mapped to the latest swine genome assembly, Sscrofa 11.1, which was constructed using Pacific Biosciences' long read sequencing technology. Reads from data sets R4 and R5 were mapped to a preliminary version of the bovine long read assembly [Genbank: NKLS00000000.1].

**Table 2:** Real data sets used in this study.

**Study**	**Species**	**SRA accession**	**DNA Ext. method^a^**	**Library kit^b^**	**Read length**	**Insert size**	**Seq. platform**
R1	Swine	SRP090776	PC, SE	TS	100	350	HiSeq2500
R2	Swine	SRP125874	PC, SE	TS	150	350	NextSeq500
R3	Swine	SRP125874	WIZ	TS-PCRF	150	550	NextSeq500
R4	Cattle	SRP076471	PC, QIA	AGI	100	350	HiSeq2500
R5	Cattle	SRP076471	PC, QIA	AGI	150	350	HiSeq2500

a *PC, pheno-chloroform extraction; SE, salt extraction; WIZ, Wizard SV96 Genomic Purification Kit; QIA, QIAamp DNA Mini kit*.

b *TS, TruSeq DNA sample prep kit; TS-PCRF, TruSeq DNA PCR-Free sample prep kit; AGI, Agilent SureSelect Target Enrichment System Kit I or Kit II*.

### Mappers compared

Three BWT-based mapping algorithms were compared in this study, BWA (version 0.7.15), Bowtie2 (version 2.2.6), and HISAT2 (version 2.1.0). Bowtie2 utilizes a BWT backtracking strategy to perform a depth-first search through a suffix trie that contains all suffixes of the reference genome, which terminates when the first alignment that satisfies specific criteria is found. Similar to Bowtie2, BWA employs a BWT backtracking strategy to search for inexact matches. However, for BWA the search is bounded by a lower limit of number of mismatches in the alignment of the reads. Hence, BWA defines a smaller search space, increasing the efficiency of the search. HISAT2 uses a backtracking strategy very similar to that of Bowtie2. However, unlike BWA and Bowtie2, it employs two different types of FM indices: a global FM index representing the entire genome, as well as numerous overlapping local FM indices for regions that collectively span the genome. The use of the overlapping local indices makes it easier to align reads that span regions covered by two global indices. Although HISAT (version 1) was originally developed to serve as an aligner for RNA sequencing data, it was expanded to align genomic DNA sequences in its second version, HISAT2. The selected mappers were run with their default parameters (Table S2), with one exception. The maximum insert size parameter (−X parameter) for the 550 bp samples was increased to 650 bp in HISAT2 and Bowtie2 from their default maximum insert size of 500 bp.

### Computational resource and time measurement

A server with 40 Intel(R) [Xeon(R) CPU E5-2660 v3 @ 2.60 GHz] processors and 128 GB of RAM running CentOS 7.3.1611 operating system was employed for the alignment jobs. In all cases, the number of threads was fixed to 24. Maximum memory consumption and time measurements were obtained using the Unix “time” command.

### Mapper robustness measurement

Aligners were evaluated using two standard performance measures, precision and recall. For a given sample and alignment method, recall (sensitivity) is the number of correctly aligned reads over the total number of reads that should have been aligned, i.e., $recall=\;\frac{\#TP}{\#TP+\#FN}$, and precision (positive predictive value) is the number of correctly aligned reads over the total number of aligned reads, i.e., $precision=\;\frac{\#TP}{\#TP+\#FP}$. Here, TP, FP, and FN denote true positive, false positive, and false negative instances, respectively. Precision and recall values were computed using the dwgsim_eval program from DWGSIM. To evaluate overall aligner performance we used the area under the precision-recall curve (PR AUC), which takes on values between 0 and 1 with larger area indicating better performance.

Additionally, the percentage of properly paired reads was used as a measure of aligner performance. Paired-end reads are generated by sequencing both ends of the fragment. A read pair is said to be properly paired if both reads were mapped in the correct orientation and the insert size was maintained. The Samtools (version 1.3; Li et al., 2009) software was employed to compute the percentage of properly paired reads in each SAM file.

## Results and discussion

### Computational resource requirement and execution time

**Figure 1:**
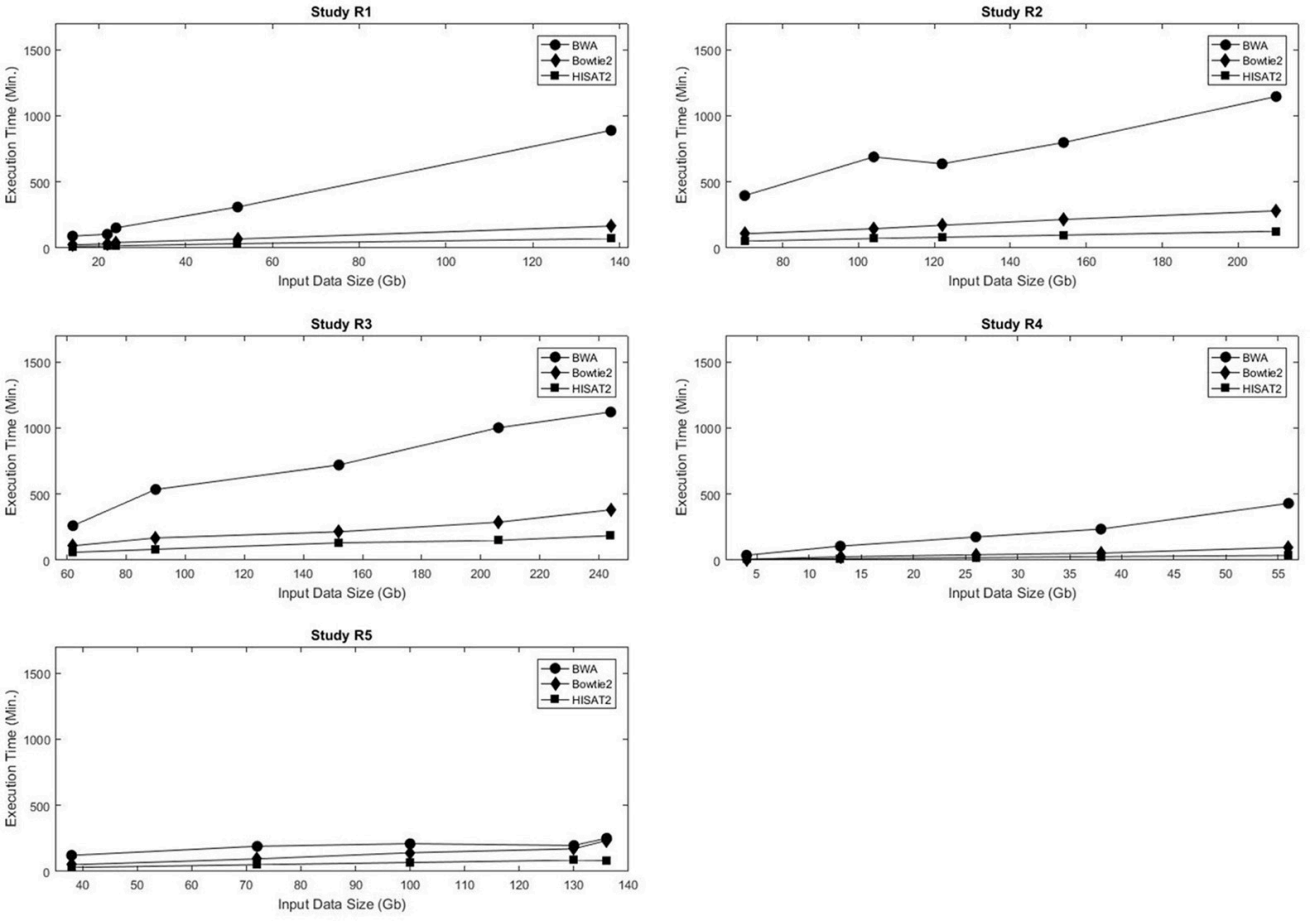
Input data size vs. execution time for the five real data sets used in this study.

Computational time and resources are critical components of the genome mapping process. Each of the three aligners being evaluated was run using 24 threads, and memory consumption and runtime were recorded. As expected, in all data sets the execution time increased with the size of the input data. For the five real data sets, the execution time of each of the aligners was linearly proportional to the size of the input data (Figure 1, Table S3). A similar trend was observed in the simulated data sets (Figures S1–S4). A comparative analysis of alignment times in the simulated data showed that, in general, HISAT2 was significantly faster than BWA and Bowtie2 (Tables S4–S9).

**Figure 2:**
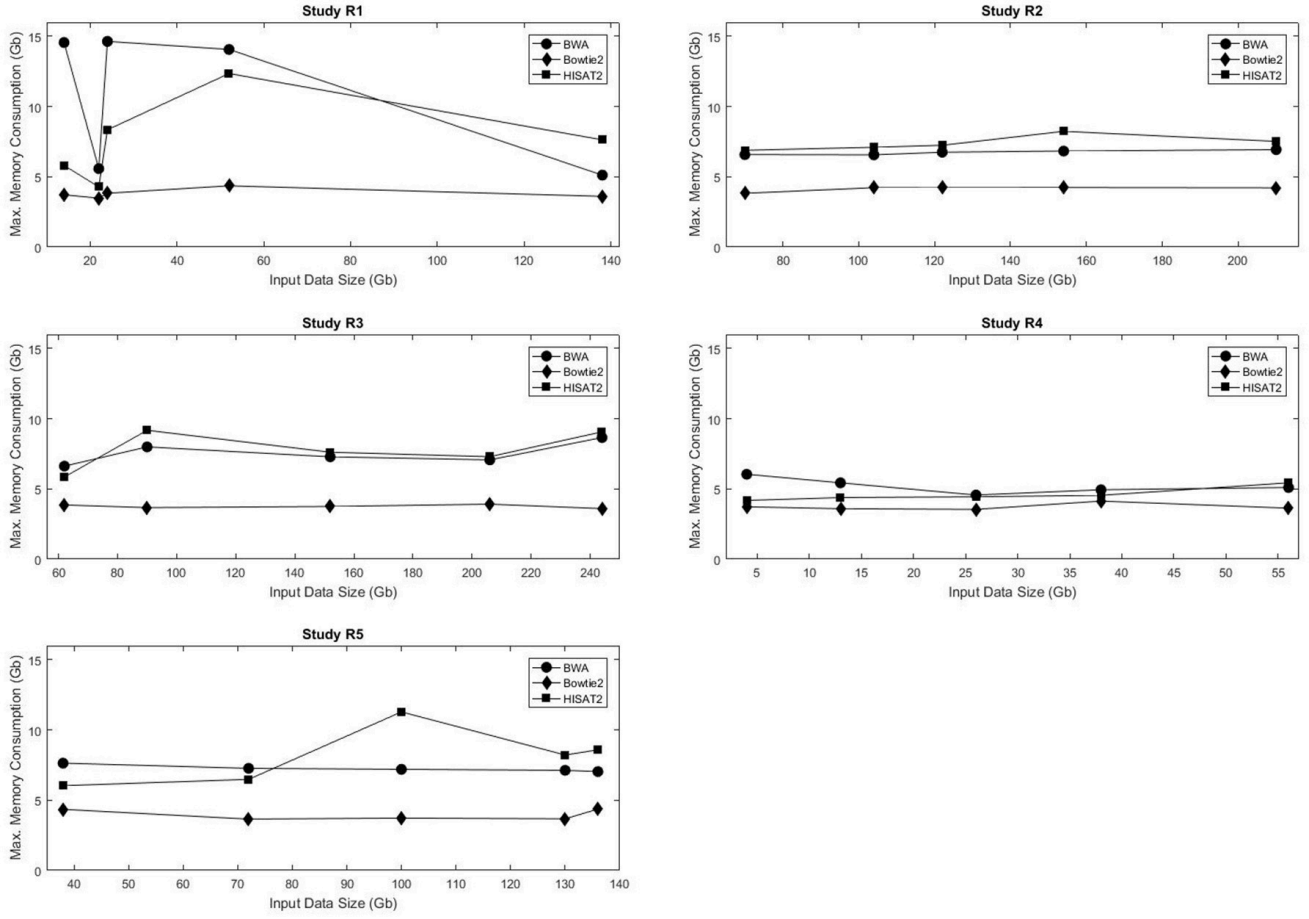
Input data size vs. execution time for the five real data sets used in this study.

Figure 2 shows the maximum memory consumption for each of the mappers in the real data sets. Unlike the execution time, memory usage was not consistently proportional to the input data size. Not only did Bowtie2 require the least amount of RAM in all data sets, but also it had the smallest variation in memory requirements, with usage ranging from 3.48 to 4.36 Gb RAM. Both BWA and HISAT2 exhibited fluctuations in memory consumption with respect to the input data size. Figures S5–S8 show the maximum RAM usage for the simulated data sets. HISAT2 utilized significantly less RAM than both BWA and Bowtie2 in all simulated sets (Tables S10–S15).

### Precision and recall of the mappers

NGS platforms provide vast quantities of data, with associated error rates ranging from 0.1 to 15% (Goodwin et al., 2016). It is essential that alignment algorithms used to map sequence data to the reference genome are able to compensate for these inherent raw data errors. Accuracy of the three mappers were assessed in our simulated data sets using the area under the precision recall curve (PR AUC), a standard performance measure.

Tables 3, 4 show the PR AUC for each mapper in the simulated 350 and 550 bp insert size data sets, respectively. All three of the mappers exhibited high PR AUC values across the 12 data sets, indicating that they are highly robust with respect to sequencing parameters. Regardless of coverage level and insert sizes, BWA demonstrated the highest performance when the read length was 100 bp, while Bowtie2 was favorable when the read length was 150 bp.

**Table 3:** Area under the precision-recall curve (PR AUC) for each mapper in each of the 350 bp insert simulated data sets.

**Data set/Mapper**	**PR AUC^a^**	**Data set/Mapper**	**PR AUC^a^**
BWA_H350_100	0.9940 (0.0109)	BWA_H350_150	0.9937 (0.0112)
Bowtie2_H350_100	0.9883 (0.0152)	Bowtie2_H350_150	0.9951 (0.0099)
Hisat2_H350_100	0.9666 (0.0254)	Hisat2_H350_150	0.9664 (0.0255)
BWA_M350_100	0.9940 (0.0109)	BWA_M350_150	0.9936 (0.0112)
Bowtie2_M350_100	0.9883 (0.0152)	Bowtie2_M350_150	0.9950 (0.0099)
Hisat2_M350_100	0.9666 (0.0254)	Hisat2_M350_150	0.9664 (0.0255)
BWA_L350_100	0.9883 (0.0152)	BWA_L350_150	0.9936 (0.0113)
Bowtie2_L350_100	0.9883 (0.0152)	Bowtie2_L350_150	0.9950 (0.0100)
Hisat2_L350_100	0.9667 (0.0254)	Hisat2_L350_150	0.9664 (0.0255)

a *Standard error is given in parentheses*.

**Table 4:** Area under the precision-recall curve (PR AUC) for each mapper in each of the 550 bp insert simulated data sets.

**Data set/Mapper**	**PR AUC^a^**	**Data set/Mapper**	**PR AUC^a^**
BWA_H550_100	0.9943 (0.0106)	BWA_H550_150	0.9938 (0.0111)
Bowtie2_H550_100	0.9875 (0.0157)	Bowtie2_H550_150	0.9947 (0.0103)
Hisat2_H550_100	0.9672 (0.0252)	Hisat2_H550_150	0.9667 (0.0254)
BWA_M550_100	0.9943 (0.0106)	BWA_M550_150	0.9939 (0.0110)
Bowtie2_M550_100	0.9874 (0.0157)	Bowtie2_M550_150	0.9947 (0.0103)
Hisat2_M550_100	0.9673 (0.0252)	Hisat2_M550_150	0.9668 (0.0253)
BWA_L550_100	0.9944 (0.0106)	BWA_L550_150	0.9939 (0.0111)
Bowtie2_L550_100	0.9875 (0.0157)	Bowtie2_L550_150	0.9947 (0.0103)
Hisat2_L550_100	0.9673 (0.0252)	Hisat2_L550_150	0.9666 (0.0254)

a *Standard error is given in parentheses*.

### Properly paired reads

**Figure 3:**
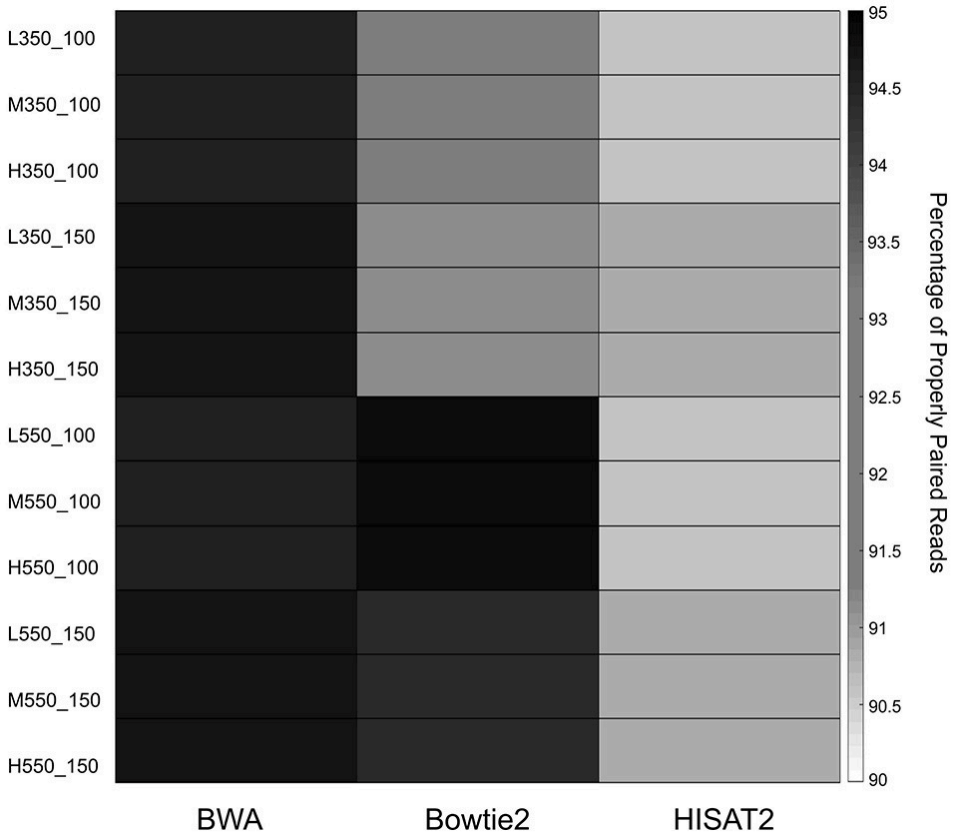
Heatmap of the average percentage of properly paired reads for the 12 simulated data sets.

The percentage of properly paired reads was used as an additional measure of performance. Paired end reads are said to be properly paired if both reads are mapped in the correct orientation with the correct insert size. Figure 3 shows the average percentage of properly paired reads identified by each of the mappers in the 12 simulated data sets. Percentages of properly paired reads ranged from 90.56 to 94.91%. BWA showed high percentages of properly paired reads across all 12 data sets, while Bowtie2 had high percentages in the 550 bp insert data sets. HISAT2 showed the lowest percentages of properly paired reads with particularly low percentages in the 100 bp read length data sets.

## Conclusion

The selection of an appropriate aligner is a crucial step in the NGS analysis process. There are various technical and biological features that can complicate the alignment process. For example, each genome has unique characteristics, such as number of chromosomes, GC content, and size. During the sequencing process there are a wide set of parameters that need to be chosen, including sequencing platform, paired-end or single-read reads, insert size, and read length. Performance of the aligners depends on all of these aspects. In this study, we evaluated the performance of three BWT-based alignment algorithms on a large mammalian genomes sequenced on the Illumina platform.

BWA and Bowtie2 demonstrated greater robustness than HISAT2. They were found to have higher PR AUC values than HISAT2, with BWA having superior values in the 100 bp read length data sets and Bowtie2 being favored in the 150 bp read length data sets. BWA exhibited strong performance in the percentage of properly paired reads across the 12 data sets. In fact, it had the highest percentage in all but the 550 bp insert, read length 100 bp data sets. Bowtie2 showed high percentages of properly paired reads in the 550 bp insert data sets.

Comparative analysis of execution time and memory consumption using the simulated data sets showed that HISAT2 was significantly faster and used less memory than both BWA and Bowtie2. However, in the 5 real data sets it was Bowtie2 that required the least amount of RAM, and it had the smallest variation in memory requirements.

All of the features described above should be taken into account when choosing an aligner. Taking the results from this study into account, Table 5 depicts an overall scoring of the aligners based on our evaluation criteria. Our results do not lead to a single mapper to be used in all scenarios but rather show that the choice of alignment tool should be driven by the application and sequencing technology.

**Table 5:** Scoring of aligners for various sequencing parameters based on criteria evaluated in this study; + indicates low score, ++ indicates medium score, and +++ indicates high score.

	**Execution time**				**Memory usage**				**Accuracy**				**% Prop. paired reads**			
Ins. (bp)	350		550		350		550		350		550		350		550	
RL (bp)	100	150	100	150	100	150	100	150	100	150	100	150	100	150	100	150
BWA	+	+	+	+	+	+	+	+	+++	+++	+++	+++	+++	+++	+++	+++
Bowtie2	++	++	++	++	+++	+++	++	++	+++	+++	+++	+++	++	++	+++	+++
HISAT2	+++	+++	+++	+++	+++	+++	+++	+++	++	++	++	++	+	+	+	+

## Author notes

The U.S. Department of Agriculture (USDA) prohibits discrimination in all its programs and activities on the basis of race, color, national origin, age, disability, and where applicable, sex, marital status, familial status, parental status, religion, sexual orientation, genetic information, political beliefs, reprisal, or because all or part of an individual's income is derived from any public assistance program. (Not all prohibited bases apply to all programs). Persons with disabilities who require alternative means for communication of program information (Braille, large print, audiotape, etc.) should contact USDA's TARGET Center at (202) 720-2600 (voice and TDD). To file a complaint of discrimination, write to USDA, Director, Office of Civil Rights, 1400 Independence Avenue, S.W., Washington, D.C. 20250-9410, or call (800) 795-3272 (voice) or (202) 720-6382 (TDD). USDA is an equal opportunity provider and employer.

## Author contributions

BK: Conceived and designed the experiment, analyzed the data, interpreted the results, and drafted the manuscript; WS: Assisted in designing the experiment and contributed to the writing of the manuscript. Both authors read and approved the final manuscript.

### Conflict of interest statement

The authors declare that the research was conducted in the absence of any commercial or financial relationships that could be construed as a potential conflict of interest.
