# Supplementary material for: Comparison of Burrows-Wheeler Transform-Based Mapping Algorithms Used in High-Throughput Whole-Genome Sequencing: Application to Illumina Data for Livestock Genomes
Source: Front Genet. 2018 Feb 26;9:35. doi: 10.3389/fgene.2018.00035 (PMC5834436; doi:10.3389/fgene.2018.00035)
Supplement: Supplementary file 4 [file Table4.DOCX]

|  | H350_100  BWA | H350_100  Bowtie2 | H350_100  HISAT2 | H350_150  BWA | H350_150  Bowtie2 | H350_150  HISAT2 |
| --- | --- | --- | --- | --- | --- | --- |
| H350_100  BWA  (SE = 1.1961) | - | 1.0 | 1.0 | - | - | - |
| H350_100  Bowtie2  (SE = 0.7796) | 1.97E-10 | - | 0.99 | - | - | - |
| H350_100  HISAT2  (SE = 0.6986) | 8.19E-15 | 1.52E-04 | - | - | - | - |
| H350_150  BWA  (SE = 1.0070) | - | - | - | - | 1.0 | 1.0 |
| H350_150  Bowtie2  (SE = 0.8048) | - | - | - | 1.96E-06 | - | 1.0 |
| H350_150  HISAT2  (SE = 0.5869) | - | - | - | 6.78E-14 | 1.45E-06 | - |
