# Supplementary material for: Comparison of Burrows-Wheeler Transform-Based Mapping Algorithms Used in High-Throughput Whole-Genome Sequencing: Application to Illumina Data for Livestock Genomes
Source: Front Genet. 2018 Feb 26;9:35. doi: 10.3389/fgene.2018.00035 (PMC5834436; doi:10.3389/fgene.2018.00035)
Supplement: Supplementary file 5 [file Table5.DOCX]

|  | M350_100  BWA | M350_100  Bowtie2 | M350_100  HISAT2 | M350_150  BWA | M350_150  Bowtie2 | M350_150  HISAT2 |
| --- | --- | --- | --- | --- | --- | --- |
| M350_100  BWA  (SE = 0.4487) | - | 1.0 | 1.0 | - | - | - |
| M350_100  Bowtie2  (SE = 0.3794) | 1.01E-11 | - | 0.64 | - | - | - |
| M350_100  HISAT2  (SE = 0.3625) | 1.04E-12 | 0.3639 | - | - | - | - |
| M350_150  BWA  (SE = 0.3672) | - | - | - | - | 1.0 | 1.0 |
| M350_150  Bowtie2  (SE = 0.3286) | - | - | - | 2.99E-10 | - | 0.9953 |
| M350_150  HISAT2  (SE = 0.2489) | - | - | - | 8.64E-15 | 0.005 | - |
