# Supplementary material for: Comparison of Burrows-Wheeler Transform-Based Mapping Algorithms Used in High-Throughput Whole-Genome Sequencing: Application to Illumina Data for Livestock Genomes
Source: Front Genet. 2018 Feb 26;9:35. doi: 10.3389/fgene.2018.00035 (PMC5834436; doi:10.3389/fgene.2018.00035)
Supplement: Supplementary file 6 [file Table6.DOCX]

|  | L350_100  BWA | L350_100  Bowtie2 | L350_100  HISAT2 | L350_150  BWA | L350_150  Bowtie2 | L350_150  HISAT2 |
| --- | --- | --- | --- | --- | --- | --- |
| L350_100  BWA  (SE = 0.3244) | - | 0.99 | 1.0 | - | - | - |
| L350_100  Bowtie2  (SE = 0.2588) | 2.52E-04 | - | 0.87 | - | - | - |
| L350_100  HISAT2  (SE = 0.2281) | 1.26E-05 | 0.1320 | - | - | - | - |
| L350_150  BWA  (SE = 0.2624) | - | - | - | - | 1.0 | 1.0 |
| L350_150  Bowtie2  (SE = 0.1700) | - | - | - | 1.18E-05 | - | 0.49 |
| L350_150  HISAT2  (SE = 0.1699) | - | - | - | 1.15E-05 | 0.52 | - |
