# Supplementary material for: Comparison of Burrows-Wheeler Transform-Based Mapping Algorithms Used in High-Throughput Whole-Genome Sequencing: Application to Illumina Data for Livestock Genomes
Source: Front Genet. 2018 Feb 26;9:35. doi: 10.3389/fgene.2018.00035 (PMC5834436; doi:10.3389/fgene.2018.00035)
Supplement: Supplementary file 7 [file Table7.DOCX]

|  | H550_100  BWA | H550_100  Bowtie2 | H550_100  HISAT2 | H550_150  BWA | H550_150  Bowtie2 | H550_150  HISAT2 |
| --- | --- | --- | --- | --- | --- | --- |
| H550_100  BWA  (SE = 1.1436) | - | 1.0 | 1.0 | - | - | - |
| H550_100  Bowtie2  (SE = 0.9328) | 8.52E-08 | - | 1.0 | - | - | - |
| H550_100  HISAT2  (SE = 0.7116) | 1.69E-15 | 1.25E-06 | - | - | - | - |
| H550_150  BWA  (SE = 0.9714) | - | - | - | - | 1.0 | 1.0 |
| H550_150  Bowtie2  (SE = 0.7594) | - | - | - | 6.55E-08 | - | 0.99 |
| H550_150  HISAT2  (SE = 0.6616) | - | - | - | 3.90E-11 | 0.0085 | - |
