# Supplementary material for: Comparison of Burrows-Wheeler Transform-Based Mapping Algorithms Used in High-Throughput Whole-Genome Sequencing: Application to Illumina Data for Livestock Genomes
Source: Front Genet. 2018 Feb 26;9:35. doi: 10.3389/fgene.2018.00035 (PMC5834436; doi:10.3389/fgene.2018.00035)
Supplement: Supplementary file 8 [file Table8.DOCX]

|  | M550_100  BWA | M550_100  Bowtie2 | | M550_100  HISAT2 | | M550_150  BWA | | M550_150  Bowtie2 | | M550_150  HISAT2 |
| --- | --- | --- | --- | --- | --- | --- | --- | --- | --- | --- |
| M550_100  BWA  (SE = 0.3488) | - | 1.0 | 1.0 | | - | | - | | - | |
| M550_100  Bowtie2  (SE = 0.2799) | 1.67E-13 | - | 0.76 | | - | | - | | - | |
| M550_100  HISAT2  (SE = 0.3289) | 5.23E-14 | 0.2420 | - | | - | | - | | - | |
| M550_150  BWA  (SE = 0.3700) | - | - | - | | - | | 1.0 | | 1.0 | |
| M550_150  Bowtie2  (SE = 0.3009) | - | - | - | | 6.44E-09 | | - | | 1.0 | |
| M550_150  HISAT2  (SE = 0.2154) | - | - | - | | 1.61E-17 | | 2.52E-09 | | - | |
