# Supplementary material for: Comparison of Burrows-Wheeler Transform-Based Mapping Algorithms Used in High-Throughput Whole-Genome Sequencing: Application to Illumina Data for Livestock Genomes
Source: Front Genet. 2018 Feb 26;9:35. doi: 10.3389/fgene.2018.00035 (PMC5834436; doi:10.3389/fgene.2018.00035)
Supplement: Supplementary file 9 [file Table9.DOCX]

|  | L550_100  BWA | L550_100  Bowtie2 | L550_100  HISAT2 | L550_150  BWA | L550_150  Bowtie2 | L550_150  HISAT2 |
| --- | --- | --- | --- | --- | --- | --- |
| L550_100  BWA  (SE = 0.2549) | - | 1.0 | 1.0 | - | - | - |
| L550_100  Bowtie2  (SE = 0.1529) | 1.24E-08 | - | 0.95 | - | - | - |
| L550_100  HISAT2  (SE = 0.1642) | 2.80E-10 | 0.05 | - | - | - | - |
| L550_150  BWA  (SE = 0.2410) | - | - | - | - | 0.98 | 1.0 |
| L550_150  Bowtie2  (SE = 0.1998) | - | - | - | 3.47E-04 | - | 0.57 |
| L550_150  HISAT2  (SE = 0.1773) | - | - | - | 1.11E-04 | 0.44 | - |
