# Supplementary material for: Comparison of Burrows-Wheeler Transform-Based Mapping Algorithms Used in High-Throughput Whole-Genome Sequencing: Application to Illumina Data for Livestock Genomes
Source: Front Genet. 2018 Feb 26;9:35. doi: 10.3389/fgene.2018.00035 (PMC5834436; doi:10.3389/fgene.2018.00035)
Supplement: Supplementary file 11 [file Table11.DOCX]

|  | M350_100  BWA | M350_100  Bowtie2 | M350_100  HISAT2 | M350_150  BWA | M350_150  Bowtie2 | M350_150  HISAT2 |
| --- | --- | --- | --- | --- | --- | --- |
| M350_100  BWA  (SE = 0. 931) | - | 8.87E-13 | 1.0 | - | - | - |
| M350_100  Bowtie2  (SE = 1.377) | 1.0 | - | 1.0 | - | - | - |
| M350_100  HISAT2  (SE = 1.367) | 3.45E-18 | 3.53E-18 | - | - | - | - |
| M350_150  BWA  (SE = 3.733) | - | - | - | - | 1.0 | 1.0 |
| M350_150  Bowtie2  (SE = 7.672) | - | - | - | 1.39E-17 | - | 1.0 |
| M350_150  HISAT2  (SE = 1.261) | - | - | - | 3.53E-18 | 3.53E-18 | - |
