# Supplementary material for: Comparison of Burrows-Wheeler Transform-Based Mapping Algorithms Used in High-Throughput Whole-Genome Sequencing: Application to Illumina Data for Livestock Genomes
Source: Front Genet. 2018 Feb 26;9:35. doi: 10.3389/fgene.2018.00035 (PMC5834436; doi:10.3389/fgene.2018.00035)
Supplement: Supplementary file 14 [file Table14.DOCX]

|  | M550_100  BWA | M550_100  Bowtie2 | M550_100  HISAT2 | M550_150  BWA | M550_150  Bowtie2 | M550_150  HISAT2 |
| --- | --- | --- | --- | --- | --- | --- |
| M550_100  BWA  (SE = 0. 847) | - | 1.22E-14 | 1.0 | - | - | - |
| M550_100  Bowtie2  (SE = 1.145) | 1.0 | - | 1.0 | - | - | - |
| M550_100  HISAT2  (SE = 1.382) | 3.41E-18 | 3.53E-18 | - | - | - | - |
| M550_150  BWA  (SE = 3.529) | - | - | - | - | 1.0 | 1.0 |
| M550_150  Bowtie2  (SE = 1.225) | - | - | - | 1.21E-15 | - | 1.0 |
| M550_150  HISAT2  (SE = 1.526) | - | - | - | 3.53E-18 | 3.53E-18 | - |
