# Supplementary material for: Comparison of Burrows-Wheeler Transform-Based Mapping Algorithms Used in High-Throughput Whole-Genome Sequencing: Application to Illumina Data for Livestock Genomes
Source: Front Genet. 2018 Feb 26;9:35. doi: 10.3389/fgene.2018.00035 (PMC5834436; doi:10.3389/fgene.2018.00035)
Supplement: Supplementary file 15 [file Table15.DOCX]

|  | L550_100  BWA | | L550_100  Bowtie2 | L550_100  HISAT2 | L550_150  BWA | L550_150  Bowtie2 | L550_150  HISAT2 |
| --- | --- | --- | --- | --- | --- | --- | --- |
| L550_100  BWA  (SE = 0.003) | - | 2.91E-11 | | 1.0 | - | - | - |
| L550_100  Bowtie2  (SE = 6.874) | 1.0 | - | | 1.0 | - | - | - |
| L550_100  HISAT2  (SE = 1.739) | 3.43E-18 | 3.53E-18 | | - | - | - | - |
| L550_150  BWA  (SE = 9.281) | - | - | | - | - | 1.0 | 1.0 |
| L550_150  Bowtie2  (SE = 3.963) | - | - | | - | 3.56E-17 | - | 1.0 |
| L550_150  HISAT2  (SE = 1.206) | - | - | | - | 3.53E-18 | 3.53E-18 | - |
