# Supplementary material for: Comparison of Burrows-Wheeler Transform-Based Mapping Algorithms Used in High-Throughput Whole-Genome Sequencing: Application to Illumina Data for Livestock Genomes
Source: Front Genet. 2018 Feb 26;9:35. doi: 10.3389/fgene.2018.00035 (PMC5834436; doi:10.3389/fgene.2018.00035)
Supplement: Supplementary file 16 [file DataSheet1.DOCX]

**Supplementary Captions**

**Table S1.** Genomic coverages for individual samples in the twelve simulated data sets.

**Table S2.** Parameters used for each of the three aligners.

**Table S3.** Execution time and memory usage for each of the three mappers in the five real data sets.

**Table S4.** Assessment of significance of differences in execution time for the 350 bp insert, high coverage simulated data sets. P-values shown compare the execution times of the row data set against the column data set using a left-tailed Wilcoxon rank-sum test. Standard error (measured in minutes) for each data set in shown in parentheses in the first column.

**Table S5.** Assessment of significance of differences in execution time for the 350 bp insert, medium coverage simulated data sets. P-values shown compare the execution times of the row data set against the column data set using a left-tailed Wilcoxon rank-sum test. Standard error (measured in minutes) for each data set in shown in parentheses in the first column.

**Table S6.** Assessment of significance of differences in execution time for the 350 bp insert, low coverage simulated data sets. P-values shown compare the execution times of the row data set against the column data set using a left-tailed Wilcoxon rank-sum test. Standard error (measured in minutes) for each data set in shown in parentheses in the first column.

**Table S7.** Assessment of significance of differences in execution time for the 550 bp insert, high coverage simulated data sets. P-values shown compare the execution times of the row data set against the column data set using a left-tailed Wilcoxon rank-sum test. Standard error (measured in minutes) for each data set in shown in parentheses in the first column.

**Table S8.** Assessment of significance of differences in execution time for the 550 bp insert, medium coverage simulated data sets. P-values shown compare the execution times of the row data set against the column data set using a left-tailed Wilcoxon rank-sum test. Standard error (measured in minutes) for each data set in shown in parentheses in the first column.

**Table S9.** Assessment of significance of differences in execution time for the 550 bp insert, low coverage simulated data sets. P-values shown compare the execution times of the row data set against the column data set using a left-tailed Wilcoxon rank-sum test. Standard error (measured in minutes) for each data set in shown in parentheses in the first column.

**Table S10.** Assessment of significance of differences in memory consumption for the 350 bp insert, high coverage simulated data sets. P-values shown compare the execution times of the row data set against the column data set using a left-tailed Wilcoxon rank-sum test. Standard error (measured in megabytes) for each data set in shown in parentheses in the first column.

**Table S11.** Assessment of significance of differences in execution time for the 350 bp insert, medium coverage simulated data sets. P-values shown compare the execution times of the row data set against the column data set using a left-tailed Wilcoxon rank-sum test. Standard error (measured in megabytes) for each data set in shown in parentheses in the first column.

**Table S12.** Assessment of significance of differences in execution time for the 350 bp insert, low coverage simulated data sets. P-values shown compare the execution times of the row data set against the column data set using a left-tailed Wilcoxon rank-sum test. Standard error (measured in megabytes) for each data set in shown in parentheses in the first column.

**Table S13.** Assessment of significance of differences in memory consumption for the 550 bp insert, high coverage simulated data sets. P-values shown compare the execution times of the row data set against the column data set using a left-tailed Wilcoxon rank-sum test. Standard error (measured in megabytes) for each data set in shown in parentheses in the first column.

**Table S14.** Assessment of significance of differences in memory consumption for the 550 bp insert, medium coverage simulated data sets. P-values shown compare the execution times of the row data set against the column data set using a left-tailed Wilcoxon rank-sum test. Standard error (measured in megabytes) for each data set in shown in parentheses in the first column.

**Table S15.** Assessment of significance of differences in memory consumption for the 550 bp insert, low coverage simulated data sets. P-values shown compare the execution times of the row data set against the column data set using a left-tailed Wilcoxon rank-sum test. Standard error (measured in megabytes) for each data set in shown in parentheses in the first column.

**Figure S1.** Genomic coverage versus execution time for the simulated 350 bp insert, 100 bp read length data sets with (A) low, (B) medium, and (C) high genomic coverage.

**Figure S2.** Genomic coverage versus execution time for the simulated 350 bp insert, 150 bp read length data sets with (A) low, (B) medium, and (C) high genomic coverage.

**Figure S3.** Genomic coverage versus execution time for the simulated 550 bp insert, 100 bp read length data sets with (A) low, (B) medium, and (C) high genomic coverage.

**Figure S4.** Genomic coverage versus execution time for the simulated 550 bp insert, 150 bp read length data sets with (A) low, (B) medium, and (C) high genomic coverage.

**Figure S5.** Genomic coverage versus maximum memory consumption for the simulated 350 bp insert, 100 bp read length data sets with (A) low, (B) medium, and (C) high genomic coverage.

**Figure S6.** Genomic coverage versus maximum memory consumption for the simulated 350 bp insert, 150 bp read length data sets with (A) low, (B) medium, and (C) high genomic coverage.

**Figure S7.** Genomic coverage versus maximum memory consumption for the simulated 550 bp insert, 100 bp read length data sets with (A) low, (B) medium, and (C) high genomic coverage.

**Figure S8.** Genomic coverage versus maximum memory consumption for the simulated 550 bp insert, 150 bp read length data sets with (A) low, (B) medium, and (C) high genomic coverage.
