# Supplementary figures and images for: Comparison of Burrows-Wheeler Transform-Based Mapping Algorithms Used in High-Throughput Whole-Genome Sequencing: Application to Illumina Data for Livestock Genomes
Source: Front Genet. 2018 Feb 26;9:35. doi: 10.3389/fgene.2018.00035 (PMC5834436; doi:10.3389/fgene.2018.00035)

**(A)**

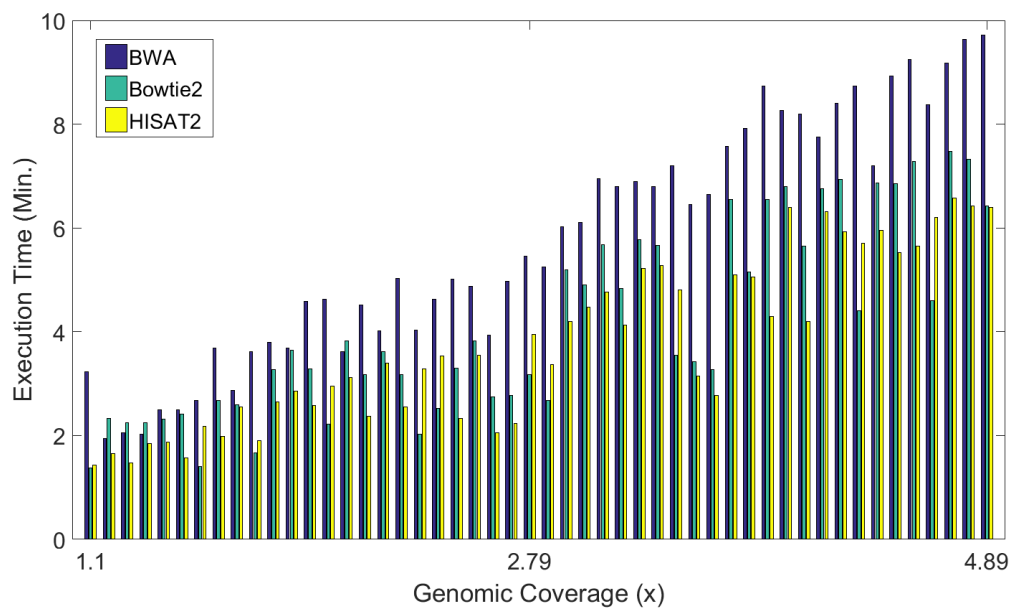

**(B)**

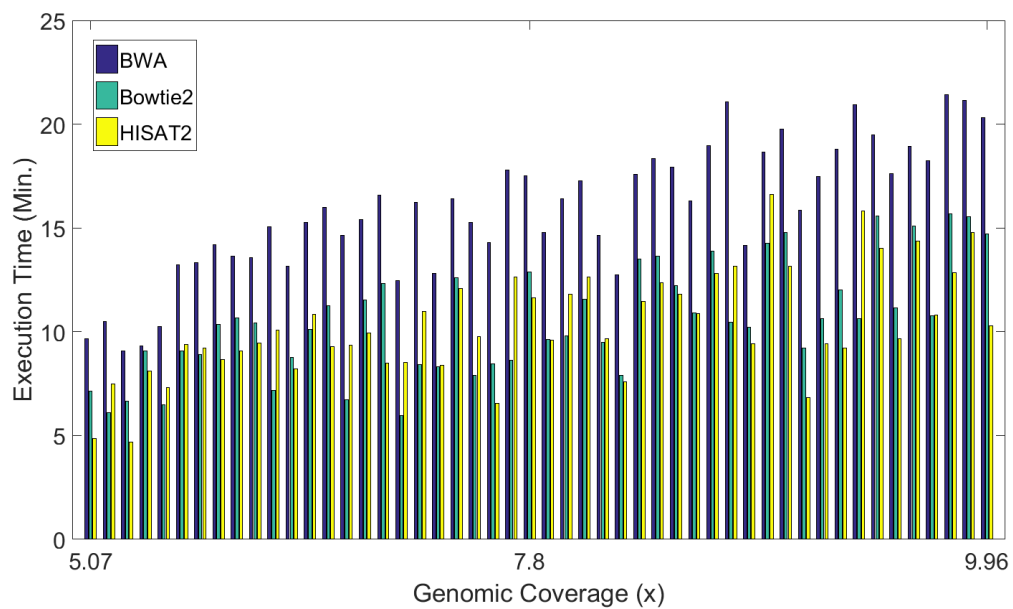

(C)

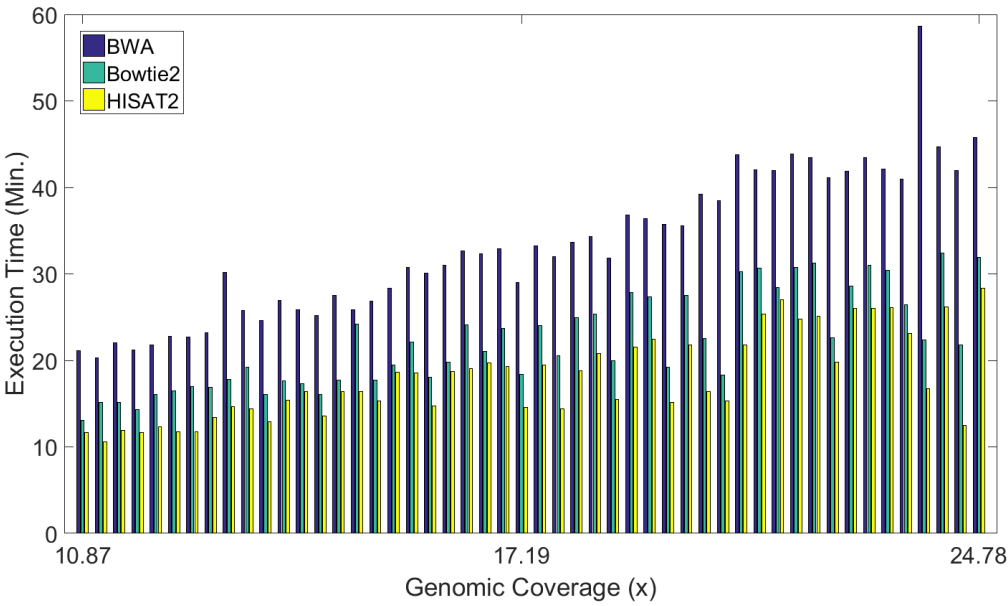

Supplement: Supplementary file 17 [file Image1.PDF]

**(A)**

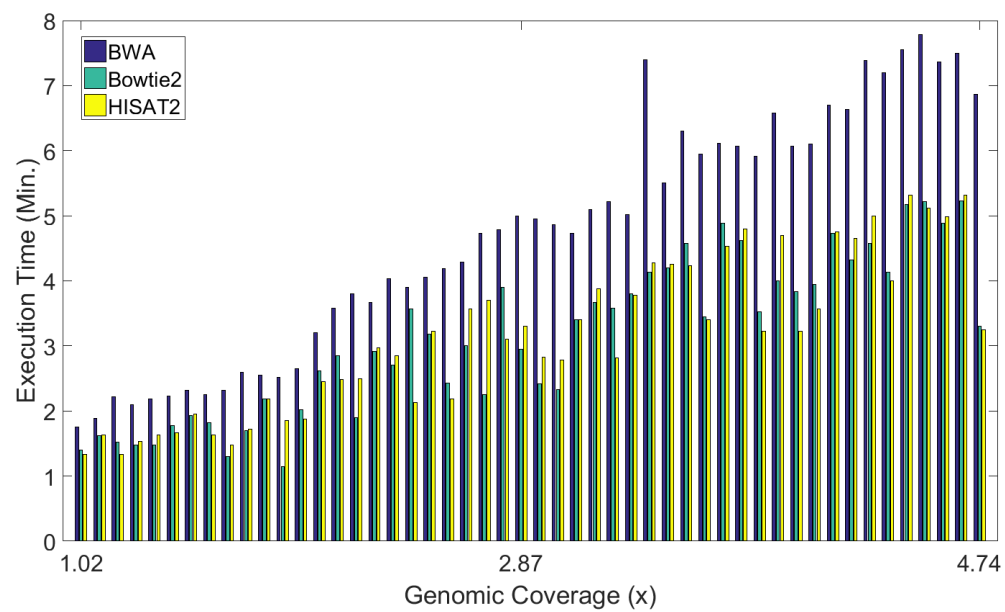

**(B)**

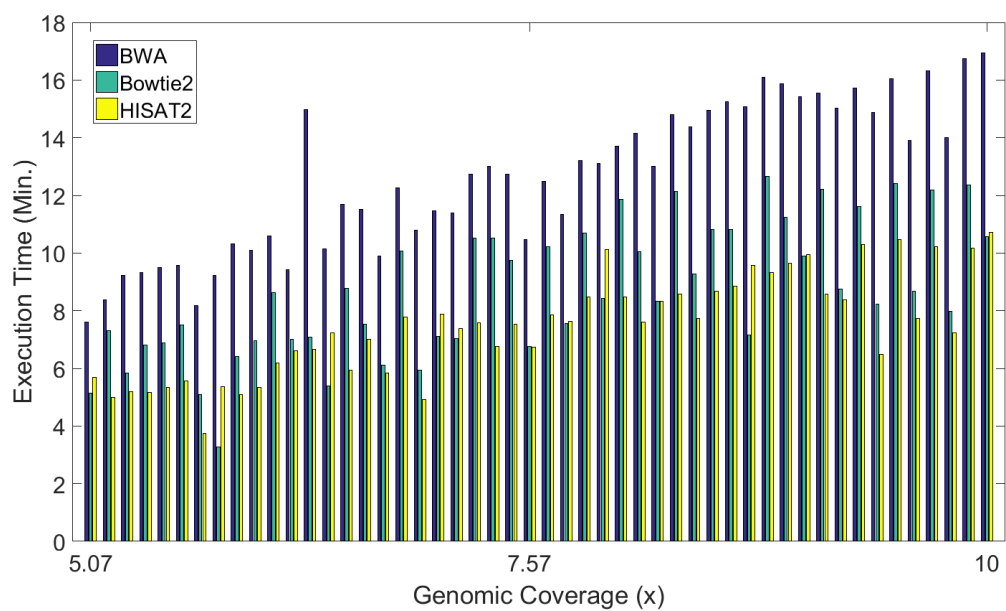

(C)

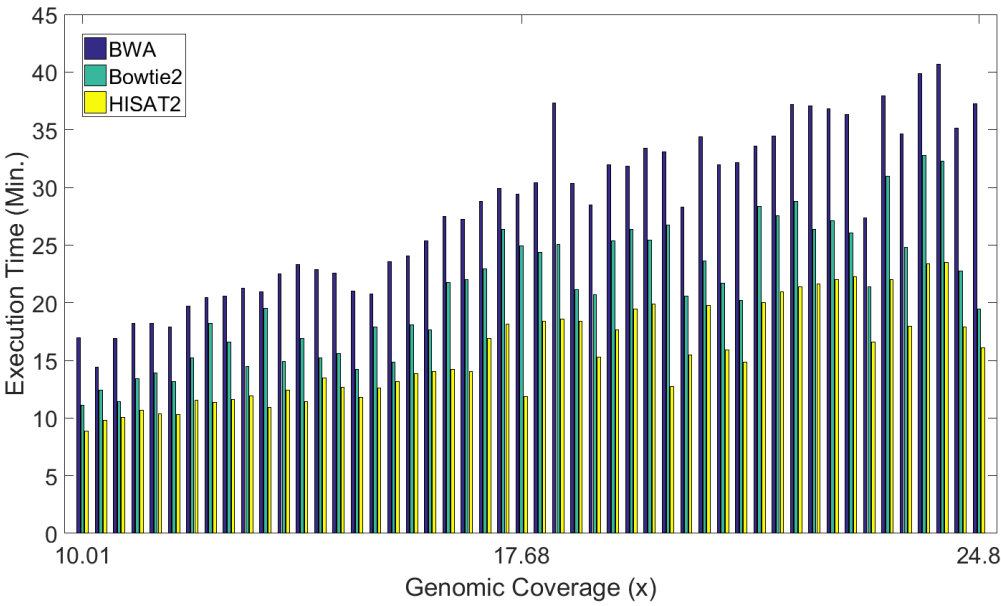

Supplement: Supplementary file 18 [file Image2.PDF]

**(A)**

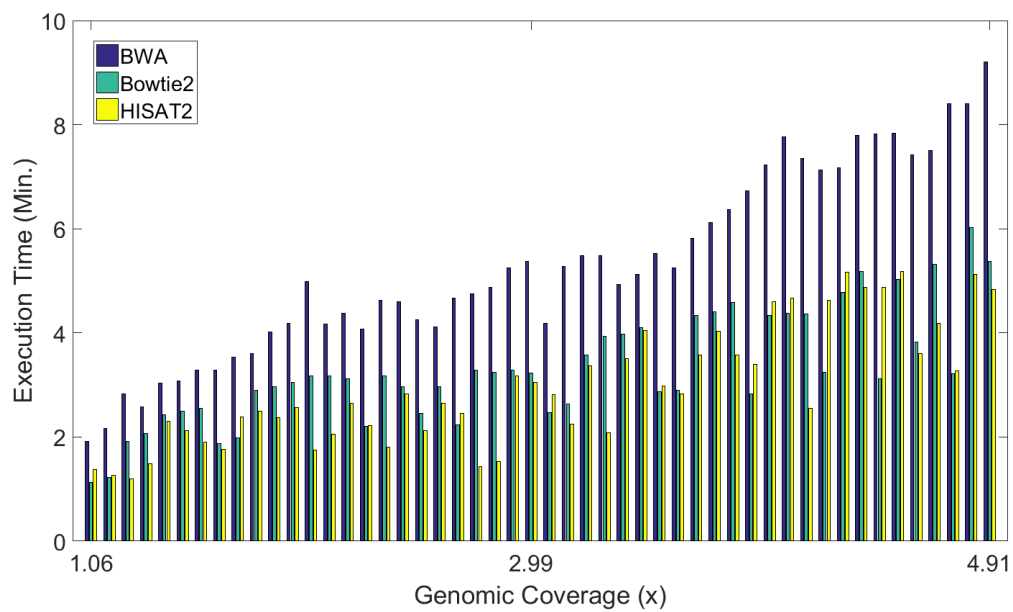

**(B)**

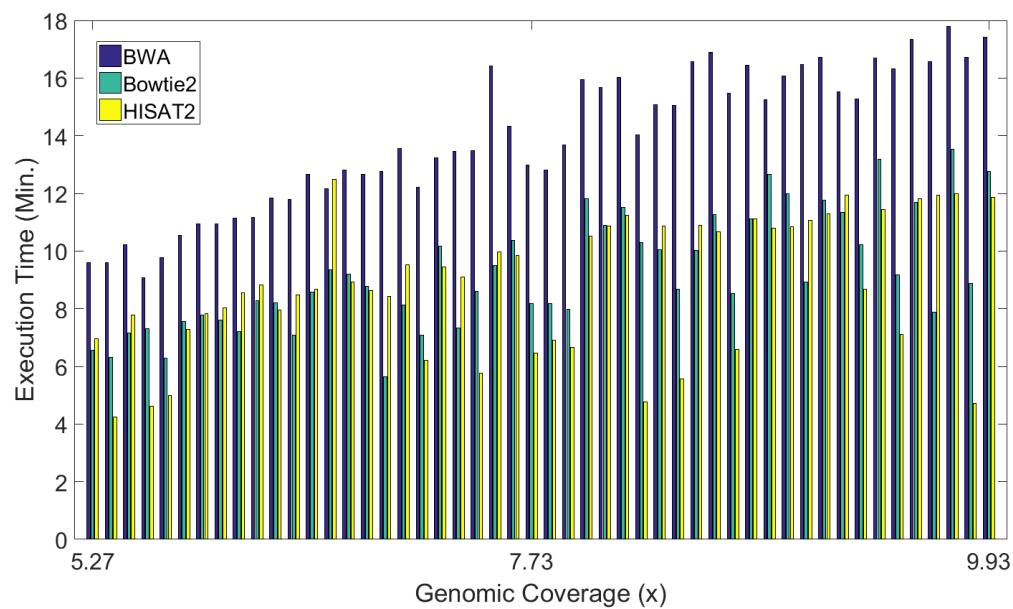

(C)

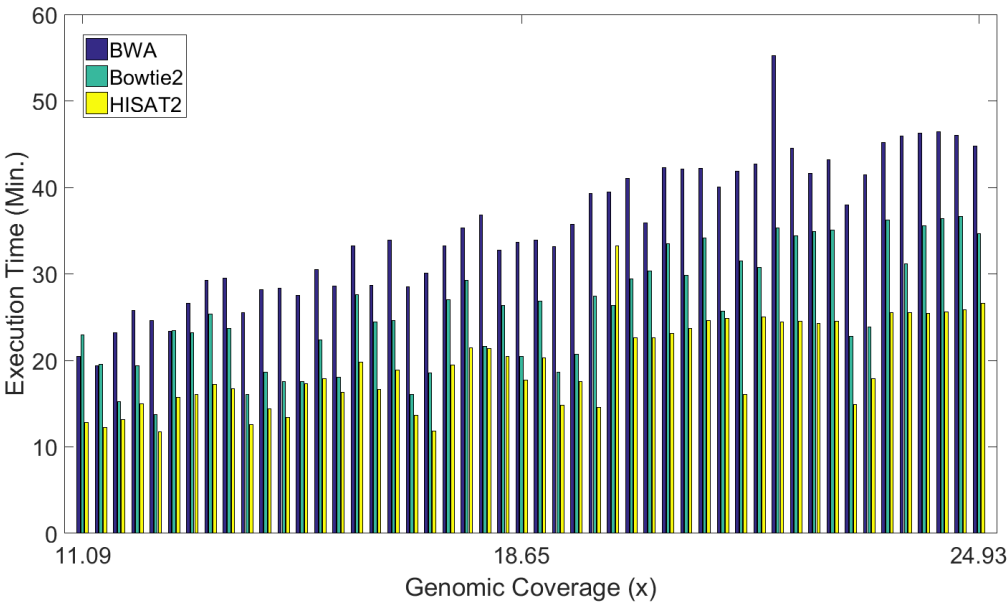

Supplement: Supplementary file 19 [file Image3.PDF]

**(A)**

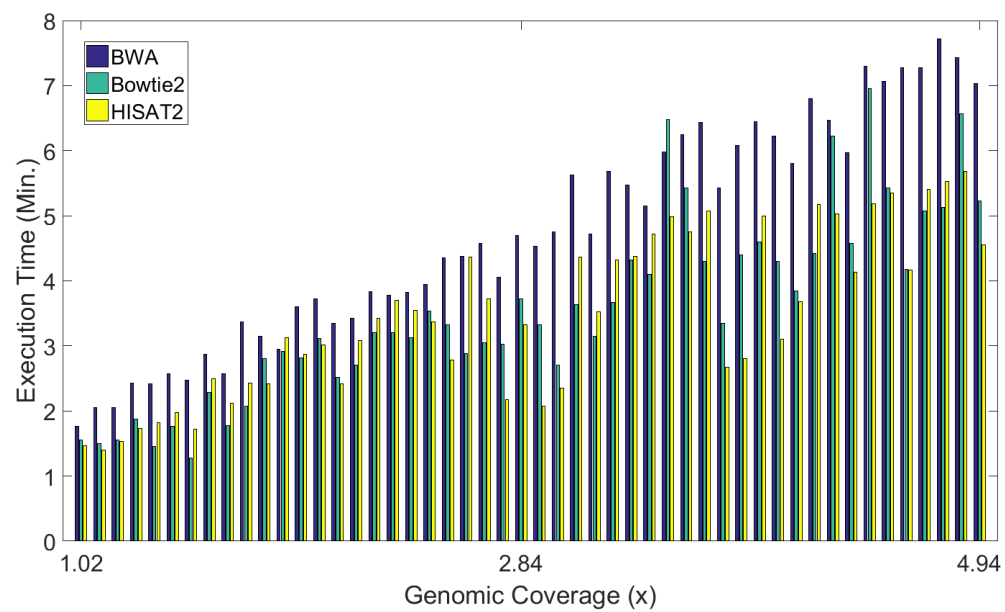

**(B)**

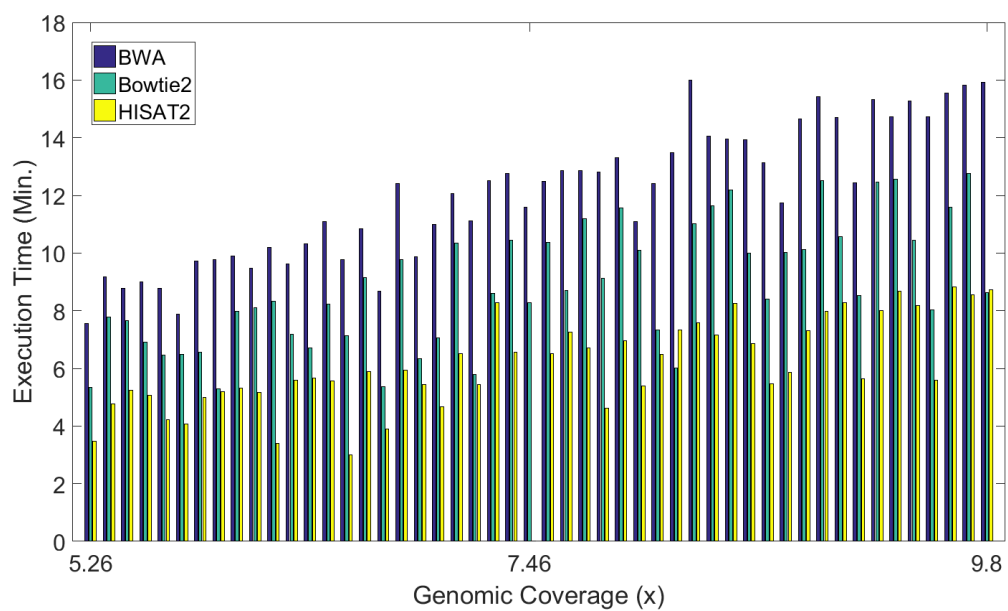

(C)

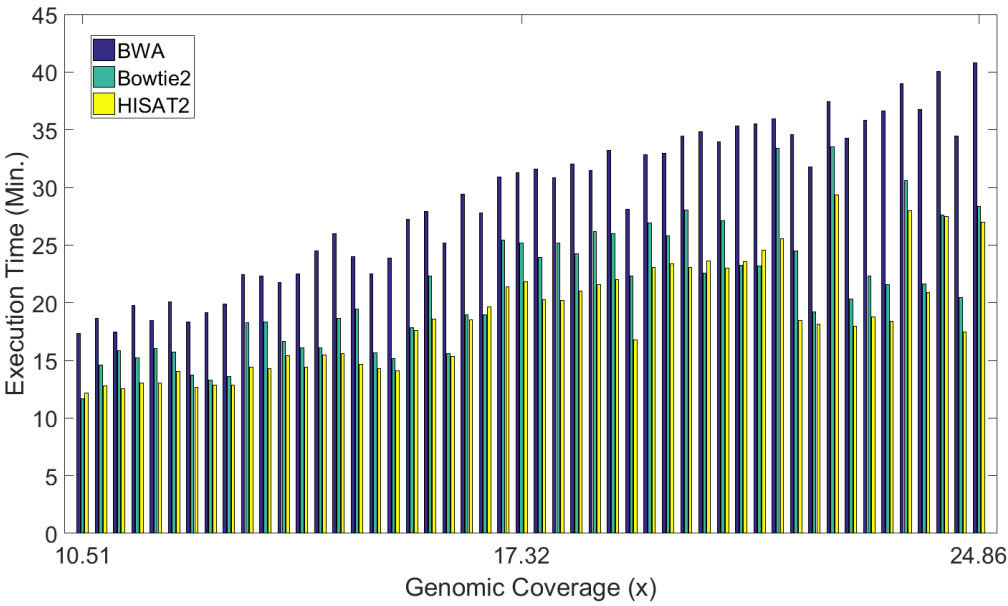

Supplement: Supplementary file 20 [file Image4.PDF]

**(A)**

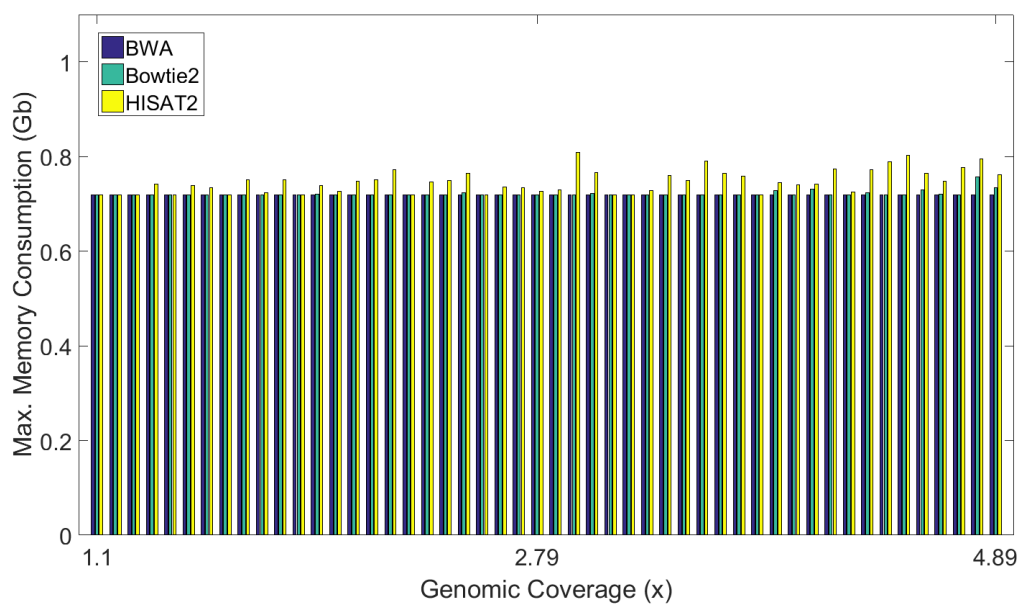

**(B)**

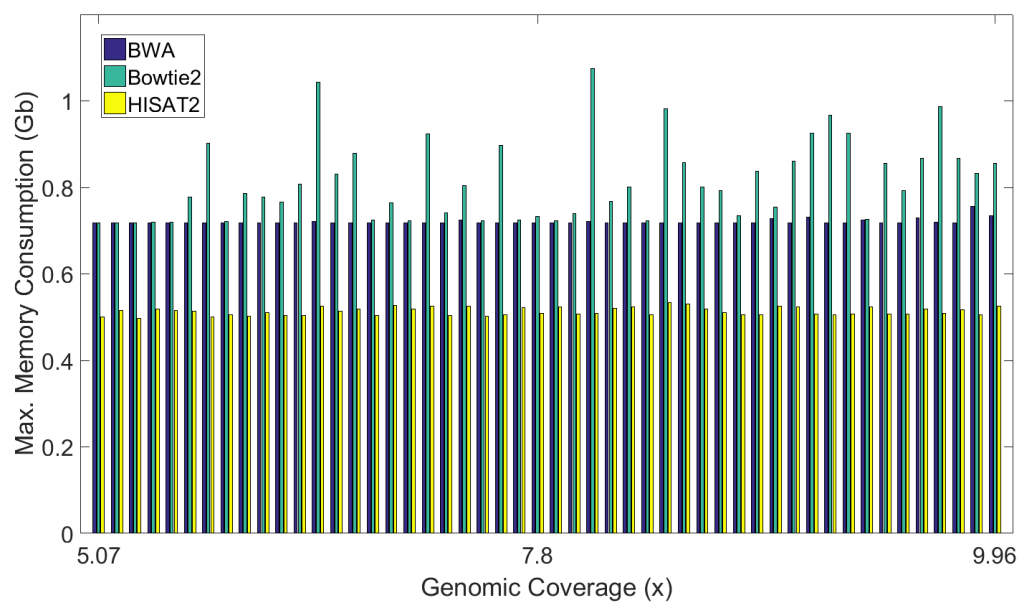

(C)

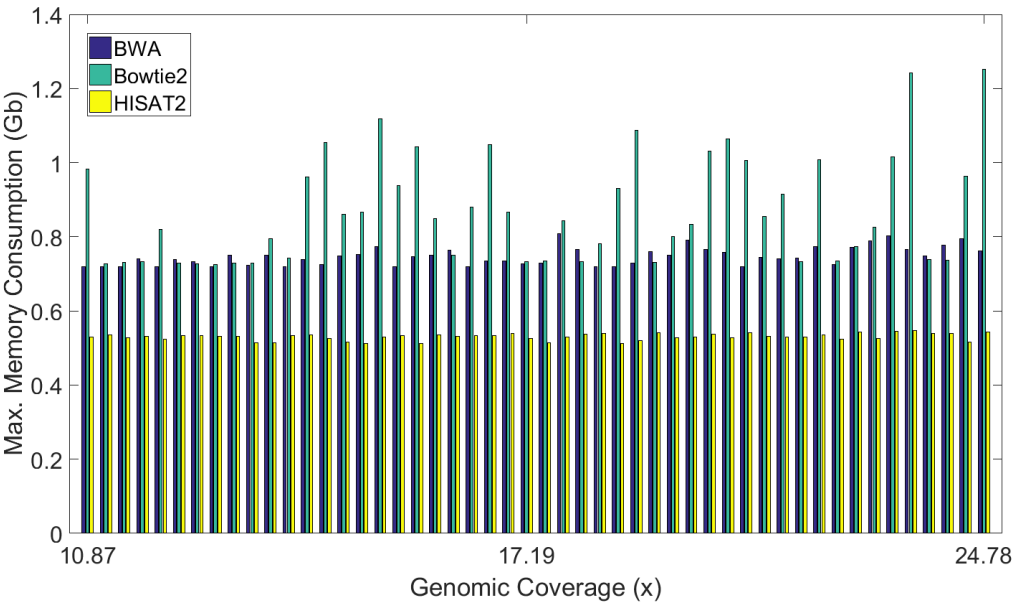

Supplement: Supplementary file 21 [file Image5.PDF]

**(A)**

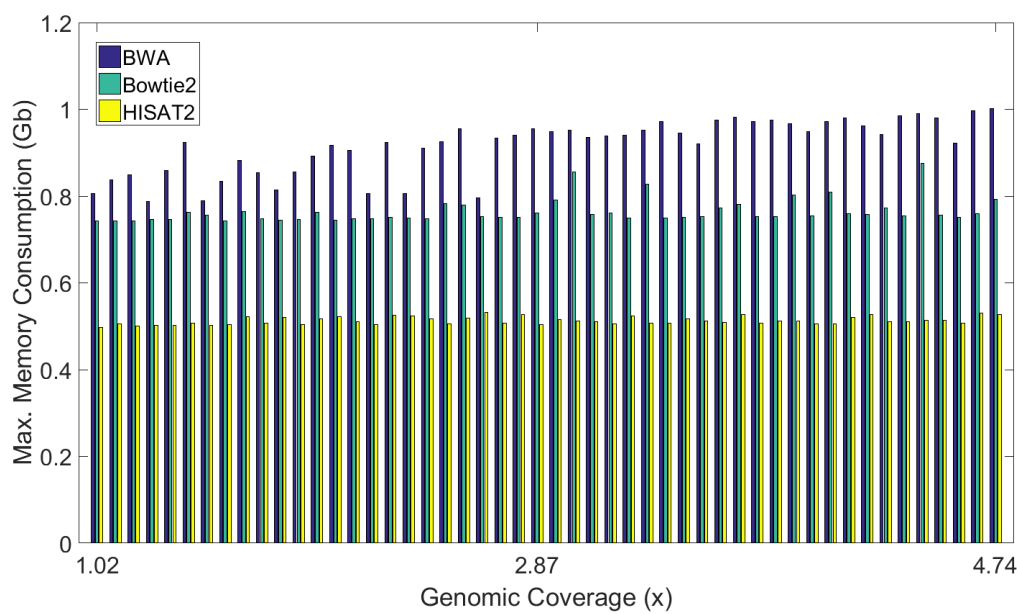

**(B)**

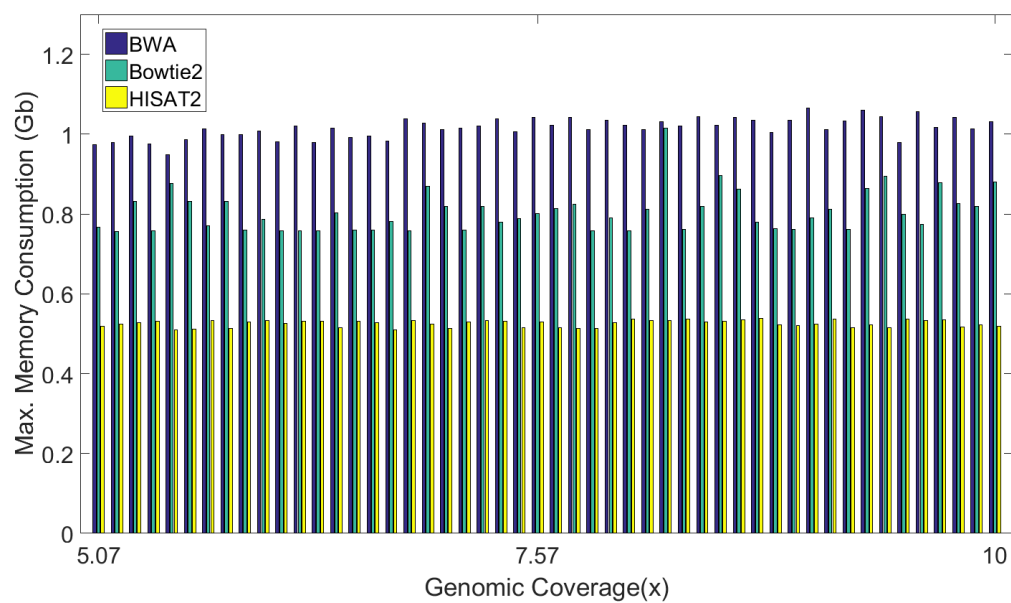

(C)

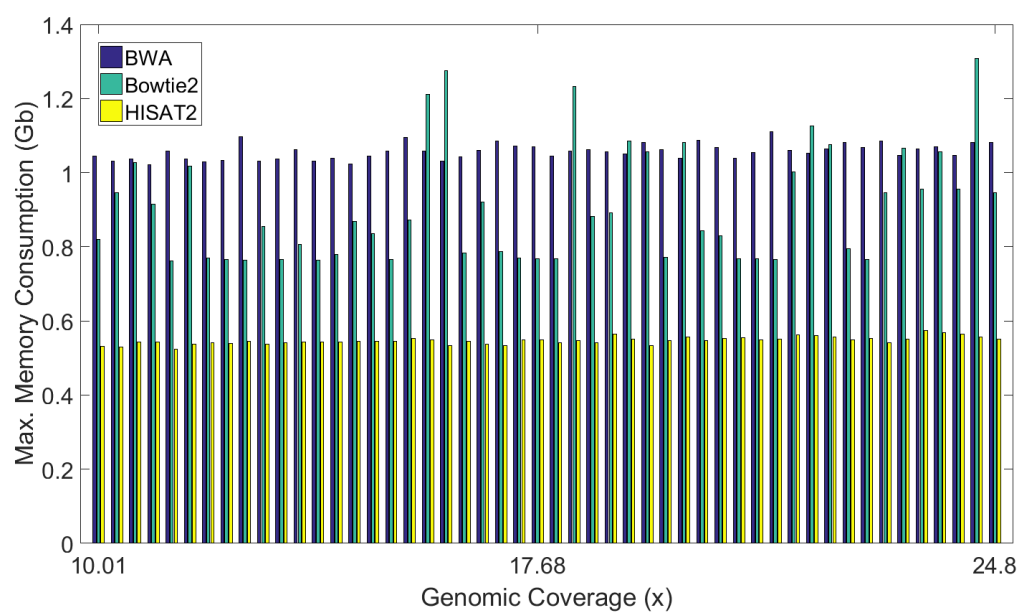

Supplement: Supplementary file 22 [file Image6.PDF]

**(A)**

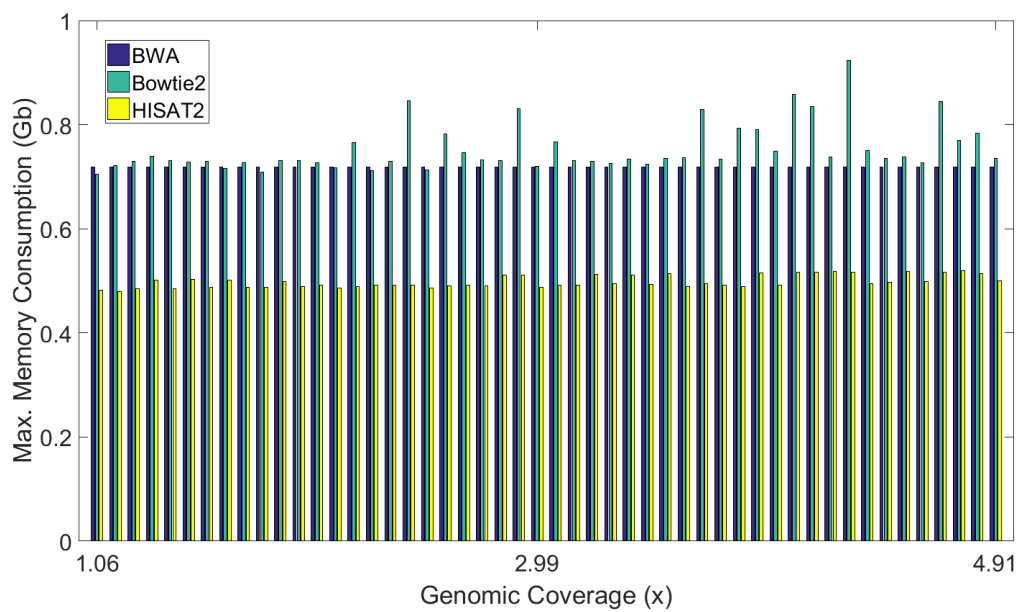

**(B)**

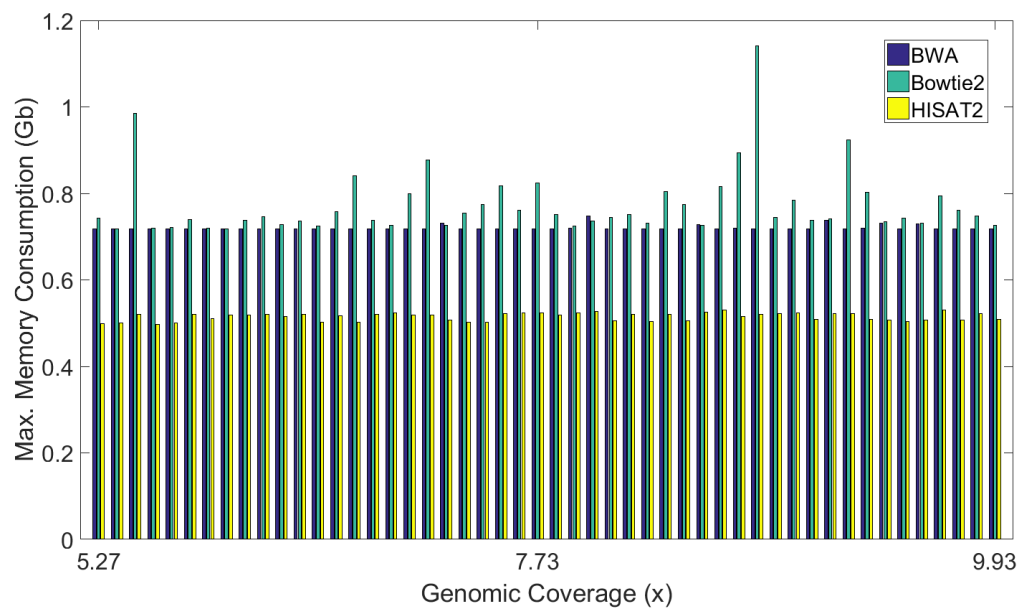

(C)

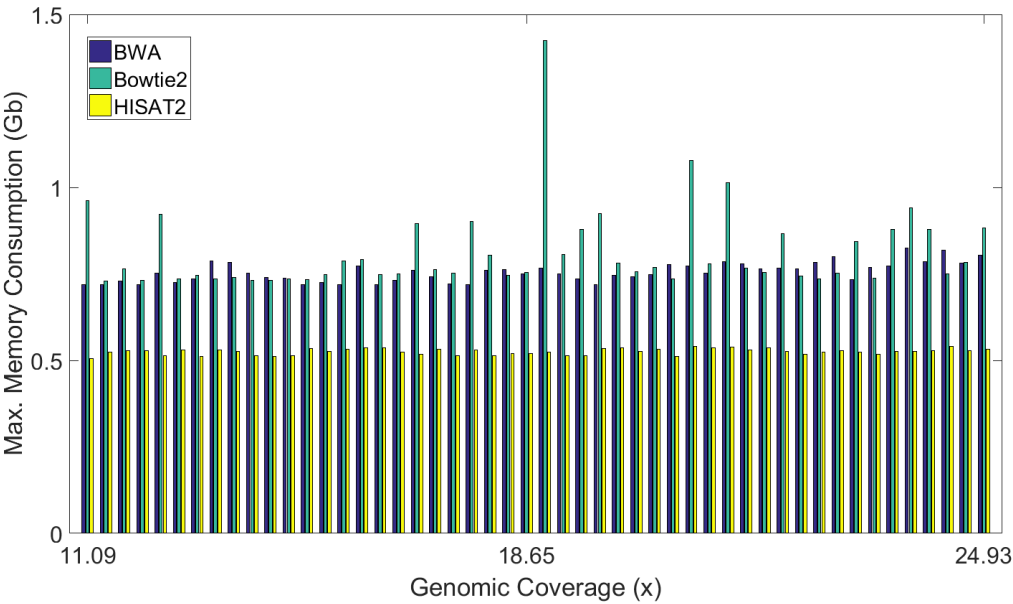

Supplement: Supplementary file 23 [file Image7.PDF]

**(A)**

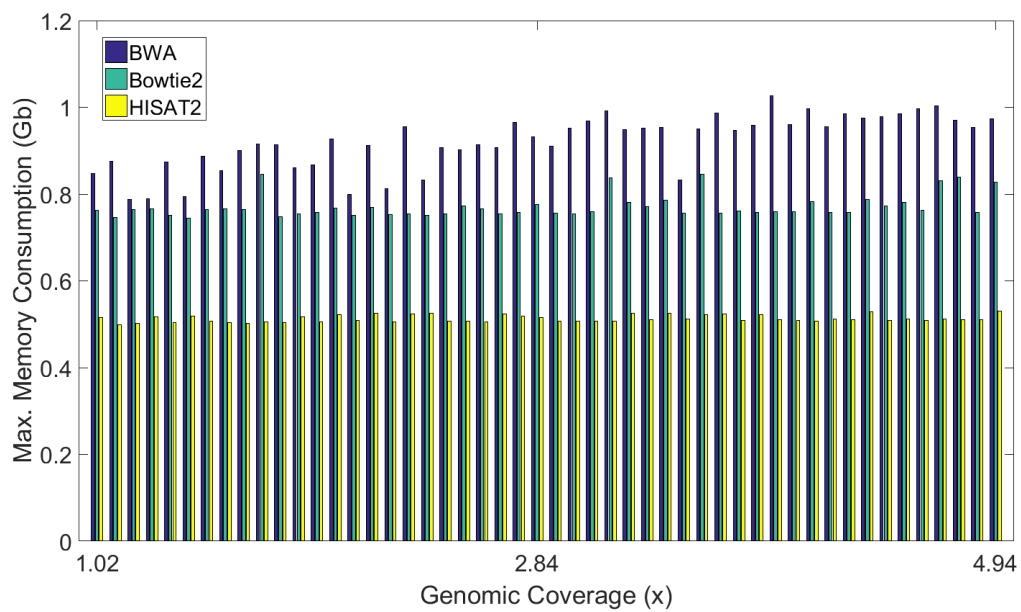

**(B)**

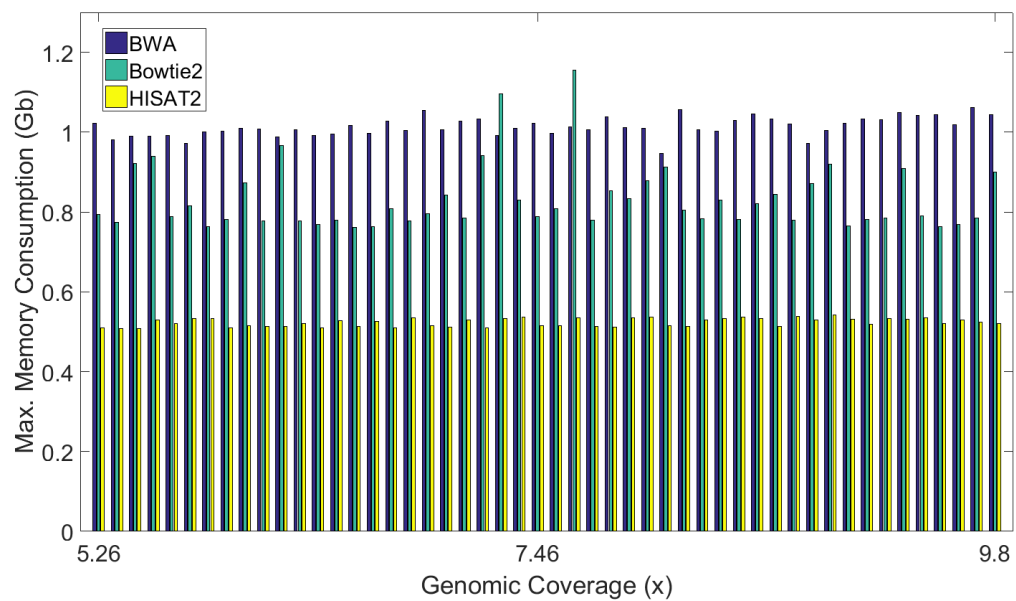

(C)

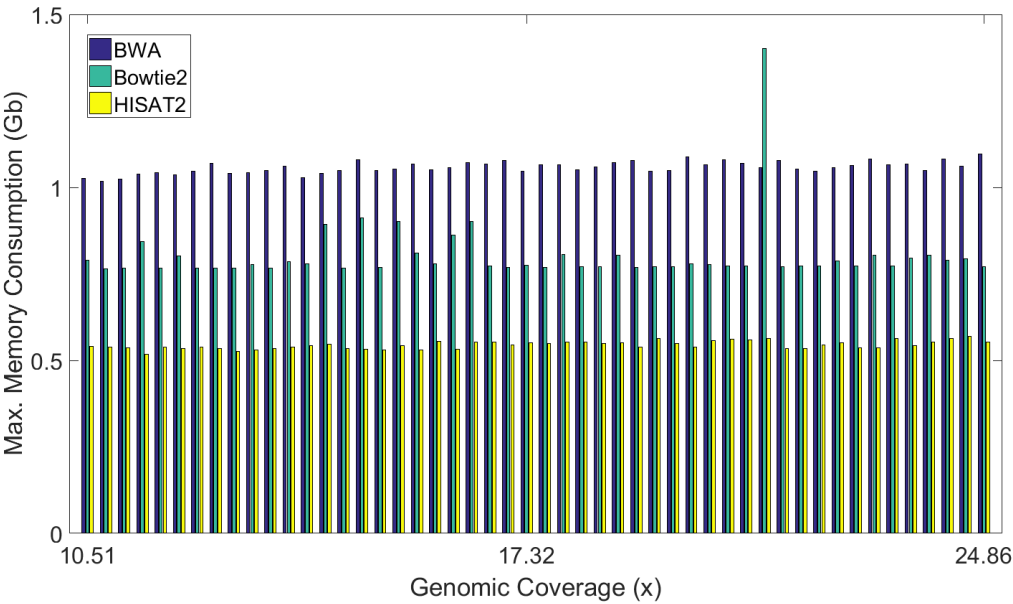

Supplement: Supplementary file 24 [file Image8.PDF]
